# Editorial: Nutrient signaling and neuroendocrine pathways in brain-organ communication

**DOI:** 10.3389/fnmol.2025.1711443

**Published:** 2025-11-25

**Authors:** Hiroyuki Aizawa, Hiroaki Masuzaki

**Affiliations:** 1 Department of Education, Aizawa Science Museum, Kasukabe, Saitama, Japan; 2 Division of Endocrinology, Diabetes and Metabolism, Hematology, Rheumatology, Graduate School of Medicine, University of the Ryukyus, Ginowan, Okinawa, Japan

**Keywords:** hypothalamus, NPY/AgRP neuron, neuroendocrine system, taste, leptin, autonomic nervous system, nutrition, mineral

Brain and internal organs communicate with one another, and nutrients mediate and modulate the communication for physical and mental health. Social stress could induce the secretion of stress hormones such as cortisol from the hypothalamic-pituitary-adrenal axis and epinephrine and norepinephrine from the sympathetic nerve ends and the adrenal medulla as physiological stress responses. The hypothalamus regulates various functions of internal organs via the autonomic nervous system, and afferent neurons innervating the organs monitor the conditions of the organs for reciprocal communication. The endocrine system also detects physical conditions to induce emotional as well as physiological reactions. For example, leptin, which is secreted from adipocytes, causes satiety in our mind and suppresses feeding behavior (Zhang et al., 1994) via the inhibition of NPY/AgRP neurons in the hypothalamus (King, 2005; Aurora, 2006). Low blood glucose causes a feeling of hunger and induces the secretion of glucocorticoids and glucagon from the adrenal cortex and pancreas, respectively, to increase the glucose level. Not only glucose but also various nutrients including fatty acids play a regulatory role in the brain and internal organs.

In this Research Topic, the authors have addressed issues concerning reciprocal communication between the brain and internal organs and its regulation by nutrients.

How does the brain control physical conditions of our internal organs according to emotions such as hunger, satiety, stress and happiness? In their original article, Wang and Mei discovered a significant positive correlation between a temporal increase in the blood glucose level and post-operative delirium after coronary artery bypass grafting in patients by a statistical analysis using the MIMIC-IV database (Johnson et al., 2023). While temporal increase in the blood glucose level was detected only in 5% of the total subjects, the positive correlation is quite significant. They had an impact on and provided inspiration to the field of post-operative delirium, followed by publications confirming that a temporal increase in blood-glucose level after an operation is a reliable marker for post-operative delirium (Lagonigro et al., 2025; Shen and Zhang, 2025). While the causal relationship between a temporal blood-glucose increase and post-operative delirium is still unclear, it is well considered that the post-operative stress that causes delirium after high-risk surgery may in some cases induce a fight-or-flight response and secrete stress hormones such as epinephrine and glucocorticoids, resulting in a temporal increase in the blood-glucose level.

How do autonomic neurons and vagal and spinal afferent neurons play a role in the communication and regulation of the brain and internal organs? Jarrah et al. reported a comprehensive review of the regulation of energy balance and metabolism by spinal afferent neurons. The vagal afferents have been identified as regulators of food intake and energy balance together with para-sympathetic neurons (Bonaz et al., 2018). Recently, spinal afferent neurons have also been revealed to play a pivotal role in the regulation of feeding behavior, nutrient sensing and energy balance (Mishra and Townsend, 2023), probably unconsciously, as well as conventional nociception, which is perceived consciously. Spinal afferent neurons may collaborate with the sympathetic neurons to regulate the innervated internal organs for physiological homeostasis and also for feeding and digestive behavior.

How do hormones and nutrients play a role in communication between the brain and internal organs for our mental and physical health? Leptin is well known to stimulate POMC neurons and inhibit NPY/AgRP neurons in the hypothalamus for the inhibition and stimulation of feeding behavior, respectively (Belgardt et al., 2009). Palmitate, which induces an unfolded protein response in the endoplasmic reticulum (ER) (Han and Kaufman, 2016), is a likely candidate to suppress the leptin signaling in POMC and NPY/AgRP neurons to cause obesity with a high-fat diet. Using 3′ mRNA-Seq transcriptome analysis, Ocłoń et al. revealed in their original article that various gene expressions have been up- or down-regulated by prolonged leptin treatment or leptin antagonist treatments, both of which should cause weight gain via leptin insensitivity in mice, on the adult mouse hypothalamic cell line mHypoA-2/12 neurons. They also demonstrated that treatments of the hypothalamic neurons with palmitate induced apoptotic cell death via the activation of caspase-3/7, presumably due to ER stress. Their gene-expression profiling study on the effects of palmitate treatment on leptin signaling in the NPY/AgRP neurons may reveal how palmitate-induced ER stress regulates leptin signaling in hypothalamic neurons.

How do nutrients stimulate and regulate the chemical sensation of gustatory papillae for taste recognition in the brain? The taste sensation, which is one of five human senses, recognizes chemical features of nutrients. There are five taste senses: umami, sweet, bitter, sour and salty. Sodium chloride, a key chemical for the salty sensation, is the most common mineral in human beings and is essential for our physiology and healthy life. Sodium ion plays a central role in the induction of action potential with voltage-gated sodium channel at axons. On the other hand, chloride ion plays an essential role in the excitation and inhibition of immature neurons and mature neurons, respectively, with GABA receptor, a chloride channel at neuronal cell body and dendrites (Moore et al., 2017). Kasahara et al. reported in their mini-review that a novel voltage-dependent chloride channel, TMC4, in taste buds is necessary for the excitation of amiloride-insensitive glossopharyngeal nerves by oral administration of salt water. Their findings clearly indicate that TMC4 plays a pivotal role in the sensation at taste buds via chloride ion flux, suggesting that further investigation of the molecular signaling and pathways for excitation of the glossopharyngeal nerves by TMC4-positive taste cells should provide a profound understanding of molecular sensing, neural processing, and perception from chemical senses to taste recognition.

Our brain senses, analyzes, perceives, and recognizes all the organs inside our body as well as the environment outside our body. We need to respond to the internal and external stimuli to maintain our body and mind healthy. Nutrient signals and neuroendocrine pathways play a central role in the regulation of the brain-organ communication for maintaining our mental and physical wellbeing.
